# Establishing contemporary trends in hepatitis B sero-epidemiology in an Indigenous population

**DOI:** 10.1371/journal.pone.0184082

**Published:** 2017-09-08

**Authors:** Jane Davies, Shu Qin Li, Steven Y. Tong, Rob W. Baird, Miles Beaman, Geoff Higgins, Benjamin C. Cowie, John R. Condon, Joshua S. Davis

**Affiliations:** 1 Department of Global and Tropical Health, Menzies School of Health Research, Darwin, Northern Territory, Australia; 2 The Infectious Diseases Department, Royal Darwin Hospital, Darwin, Northern Territory, Australia; 3 Health Gains Planning Branch, Northern Territory Department of Health, Darwin, Northern Territory, Australia; 4 Doherty Institute for Infection and Immunity, University of Melbourne, Melbourne, Victoria, Australia; 5 Victorian Infectious Diseases Service, Royal Melbourne Hospital, Melbourne, Victoria, Australia; 6 Territory Pathology, Royal Darwin Hospital, Darwin, Northern Territory, Australia; 7 Western Diagnostic Pathology, Myaree, Western Australia, Australia; 8 University of Western Australia, Perth, Western Australia, Australia; 9 University of Notre Dame Australia, Fremantle, Western Australia, Australia; 10 SA Pathology, Adelaide, South Australia, Australia; 11 WHO Collaborating Centre for Viral Hepatitis, Doherty Institute, Melbourne, Victoria, Australia; 12 John Hunter Hospital, New Lambton Heights, New South Wales, Australia; University of Cincinnati College of Medicine, UNITED STATES

## Abstract

**Background:**

Indigenous populations globally are disproportionately affected by chronic hepatitis B virus (HBV) infection however contemporary sero-prevalence data are often absent. In the Indigenous population of the Northern Territory (NT) of Australia the unique C4 sub-genotype of HBV universally circulates. There are no studies of the sero-prevalence, nor the impact of the vaccination program (which has a serotype mismatch compared to C4), at a population-wide level.

**Methods:**

We examined all available HBV serology results obtained from the three main laboratories serving NT residents between 1991 and 2011. Data were linked with a NT government database to determine Indigenous status and the most recent test results for each individual were extracted as a cross-sectional database including 88,112 unique individuals. The primary aim was to obtain a contemporary estimate of HBsAg positivity for the NT by Indigenous status.

**Results:**

Based on all tests from 2007–2011 (35,633 individuals), hepatitis B surface antigen (HBsAg) positivity was 3·40% (95%CI 3·19–3·61), being higher in Indigenous (6·08%[5·65%-6·53%]) than non-Indigenous (1·56%[1·38%-1·76%]) Australians, p<0·0001.

Birth cohort analysis showed HBsAg positivity fell over time for Indigenous people, with this decrease commencing prior to universal infant vaccination (which commenced in 1990), with an ongoing but slower rate of decline since 1990, (0·23% decrease per year versus 0·17%).

**Conclusions:**

HBsAg positivity is high in the NT, particularly in the Indigenous population. HBsAg positivity has fallen over time but a substantial part of this decrease is due to factors other than the universal vaccination program.

## Introduction

Indigenous populations worldwide, irrespective of the income level of their country of residence, are disproportionately affected by chronic hepatitis B virus (HBV) infection [1,2]. Thus Indigenous populations are heavily burdened with the sequelae of chronic liver disease and hepatocellular carcinoma [3]. The Indigenous population of the Northern Territory (NT) of Australia share many demographic features with other Indigenous populations. Indigenous Australians constitute 30% of the NT population, with many living in remote, isolated communities that are characterised by poverty and limited access to adequate health care. In the face of multiple competing health priorities including a growing burden of non-communicable chronic diseases, robust longitudinal data on HBV prevalence is required to guide the prioritisation of funding and resource allocation for hepatitis B. The World Indigenous Peoples Conference on Viral Hepatitis noted in the “*Anwernekenhe consensus statement*”[4] in 2014 that such data to inform action are desperately lacking.

Prevalence estimates for chronic HBV infection for NT Indigenous Australians range from 0·8% to 13·3% [5,6]. These estimates are based on either: small community-specific studies,[5,6] screening of antenatal women, [7,8] or Australia-wide mathematical modelling [9]. There are therefore important knowledge gaps about the epidemiology of chronic HBV infection in Indigenous Australians, particularly in males, older people and children.

The NT introduced universal infant HBV vaccination for Indigenous children in 1988 and for all children in 1990 with a catch-up program for those aged 6–16 years in 1998–9. A study using NT government notification data and midwifery registers showed a decrease in the prevalence of chronic HBV infection in females since the introduction of universal vaccination, but ongoing substantial disparity between Indigenous and non-Indigenous women [10].

We have recently determined that HBV sub genotype C4 (HBV/C4) is the unique prevalent HBV strain in Indigenous NT residents. HBV/C4 has genetic markers of an aggressive phenotype and has a different serotype to the currently used vaccine [11]. In Gambia where a serotype mismatch has also been identified, protection against HBV infection (defined as remaining negative to hepatitis B core antibody, anti-HBc) was 67% 15 years after vaccination, although protection against chronic infection (remaining HBsAg negative) was high at 96.6% [12]. Concerns arising from the findings that fully vaccinated adolescents in an NT community setting demonstrated serological evidence of both active and past HBV infection, mean that reliable sero-prevalence data are particularly important in guiding health policy [6].

This study aimed to use the results of all existing HBV serology tests from NT residents to provide a low cost, contemporary estimate of chronic HBV infection prevalence in the NT, as well as investigating associations with geographical distribution, Indigenous status and age group.

## Methods

This study was a retrospective analysis of all available HBV laboratory test results including: HBsAg, anti-HBs, and anti-HBc carried out for any reason in the NT between January 1991 and December 2011. The primary aim was to obtain an estimate of the prevalence of HBsAg positivity for the NT overall by Indigenous status. Secondary aims were to establish the prevalence of anti-HBs positivity and anti-HBc positivity, and to evaluate all HBV markers by birth cohort with reference to key dates in the introduction of universal childhood HBV vaccination in the NT.

Ethical approval was obtained from the Human Research Ethics Committee of the Northern Territory Department of Health and Menzies School of Health Research (HREC-2012-1745) and the Central Australian Human Research Ethics Committee (HREC-14-244).

### Raw data sources

It is known that 97·9% of all HBV notifications in the NT come from one of three pathology providers [13]. We therefore approached these laboratories for all HBV test results (positive and negative) for 1991–2011. Demographic details including surname, forename, birth date, gender, and community of usual residence were obtained from the pathology providers.

The data were then edited into a uniform format and amalgamated to create a master dataset where each line of data was given a unique test identification number and represented a single testing episode on a specific day (this could include more than one test result, e.g. HBsAg and anti-HBs done on the same blood draw).

### Data linkage

Individuals may have had multiple tests both over time and at different locations. Furthermore, testing data did not include Indigenous status. To identify all tests for each person and to obtain Indigenous status, we linked the testing dataset to the NT Department of Health’s Client Master Index (CMI). The CMI is a central identification module for the clinical information systems of all NT government health services. It includes name, date of birth, sex, usual place of residence, Indigenous status and a unique client identification number. Audits of the CMI have documented a high degree of accuracy [14] and it has been used in previous data linkage projects [10]. Demographic data from the testing dataset were deterministically matched to the CMI based on 11 combinations of the variables forename, surname, date of birth and address, to assign a unique person identifier to each testing record and to obtain Indigenous status for each individual. Between 5% and 50% of the data matched on each of the 11 levels was manually checked for accuracy. These demographic data were then de-identified and linked (using the unique test identification number) back to the hepatitis B test results.

### Data set for analysis

**Fig 1:**
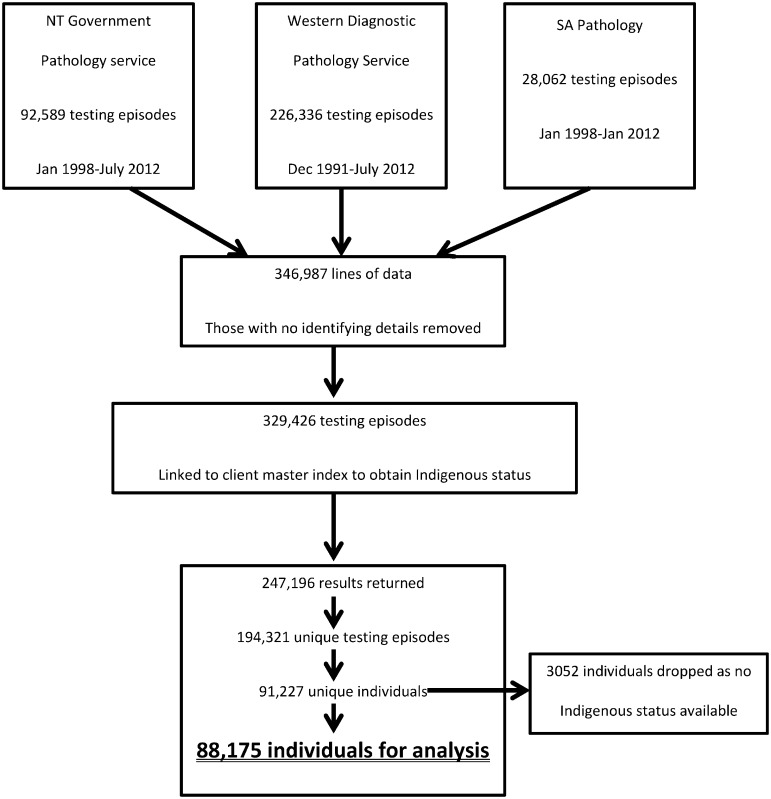
Flow diagram detailing sources of testing data and production of dataset including only the latest set of results for each individual with all the duplicate tests and individuals removed.

The dataset was then organised to include the most recent available result for each of the following tests: HBsAg, anti-HBs, and anti-HBc for each individual for whom Indigenous status was available (Fig 1).

We defined chronic HBV infection as any documented HBsAg positive result i.e. we did not require each individual to have 2 positive HBsAg test results separated by at least 6 months in time as per the strict definition of chronic hepatitis B. In order to reach this definition we conducted a sensitivity analysis using three subsets of the data: (one) the whole dataset from 1991–2011 defining chronic HBV infection as any positive HBsAg result; (two) data from 2007–2011 defining chronic HBV infection as above; and (three) data from 2007–2011 defining chronic HBV infection as two positive HBsAg results at least six months apart. This was done to assess possible underestimation of chronic HBV infection using the formal definition and to establish if there were large difference in the results obtained by each method (S1 Appendix). Guided by the sensitivity analysis we decided to focus on analysis of dataset two as described above to minimise the biasing factors such as death, loss of HBsAg and movement out of the NT likely to occur more frequently over a 21 year period.

Data were managed and analysed in STATA (Statacorp, College Station, Texas) version 13. In descriptive analysis, measures of central tendency and dispersion used were: median and inter-quartile range (IQR) for non-normally distributed variables; and mean and standard deviation (SD) for normally distributed variables. Proportions within categorical groups were calculated with the denominator as the total number of individuals who had a result available for that variable, with binomial confidence intervals. Chi squared tests were used to assess differences between categorical groups and two-way tests of proportions for differences within and across categories. Logistic regression was used for multivariable analysis of dichotomous dependent variables such as HBsAg; post estimation diagnostics included classification statistics and the Pearson Goodness of Fit test. Interrupted time series analysis using regression with Newey West standard errors was used to analyse HBsAg prevalence before and after the implementation of universal vaccination in the NT. The Cumby-Huizinga general test for auto-correlation was applied to the time series analysis to determine the appropriate lag to account for auto-correlation [15].

## Results

The study describes the most recent HBV serology results for 35,633 individuals (14,025 who identified as Indigenous Australians) who had blood collected in the NT of Australia between 2007 and 2011 inclusive (dataset two as described above). This was 15% of the total NT estimated resident population in 2011 and 20% of the NT Indigenous population [16] (see Table 1 for breakdown by age, sex and Indigenous status).

**Table 1:** Numbers of people who had one or more hepatitis B serology markers tested between 2007 and 2011 inclusive and the proportion of the NT population they represent detailed by Indigenous status and age group at the time of testing.

Age group	Indigenous						Non-Indigenous					
	Male			Female			Male			Female		
	tested	ERP	Proportion of ERP tested (%)	tested	ERP	Proportion of ERP tested (%)	tested	ERP	Proportion of ERP tested (%)	tested	ERP	Proportion of ERP tested (%)
**Under 10 years**	78	7924	1·0	71	7326	1·0	82	10678	0·8	75	10139	0·7
**10–19 years**	996	7290	13·7	1542	6829	22·6	493	9973	4·9	863	8571	10·1
**20–29 years**	1880	6452	29·1	2233	6423	34·8	1994	15562	12·8	4867	12871	37·8
**30–39 years**	1451	4869	29·8	1400	5015	28·1	2195	14422	15·2	4146	13108	31·6
**40–49 years**	1110	3873	28·7	1105	4135	26·7	1723	13581	12·7	1513	12104	12·5
**50–59 years**	605	2498	24·2	715	2602	27·5	1209	11989	10·1	1077	10500	10·3
**60–69 years**	270	1079	25·0	312	1279	24·4	592	7596	7·8	385	5515	7·0
**Over 70 years**	104	494	21·1	146	762	19·2	210	3203	6·6	150	2630	5·7
**Overall**	6494	34479	18.8	7524	34371	21.2	8498	87004	9.8	13076	75438	17.3

ERP = Estimated Resident Population from the ABS 2011 census data (16) 41 of 35633 individuals not included in this table as they either had DOB or sex missing

The majority of individuals (82·1%) were aged between 20 and 59 years at the time of testing. The median age of those tested was 32 years (IQR 25–44), with Indigenous people younger than non-Indigenous (Table 2). Females accounted for 57·8% of those tested and 39·4% of all individuals tested were Indigenous. HBsAg, anti-HBc and anti-HBs positivity by Indigenous status and sex are presented in Table 2.

**Table 2:** Demographics characteristics and HBsAg, anti-HBc and anti-HBs positive results, NT residents tested for HBV in 2007–2011, by Indigenous status and sex.

	Indigenous	Non-Indigenous	P value
**Number tested**^1^	14,025	21,608	
** Age (years) median (IQR)**	30·8 (21·5–43·3)	33.2 (26·3–44·0)	<0·001
** Female n(%)**	7531 (53·7)	7174 (60·5)	<0·001
**HBsAg**			
** Number tested**	11,730	17,123	
** Proportion positive n(%)**	713 (6·08)	267 (1·56)	<0·001
** Males n(%)**	431 (8·27)	137 (2·22)	<0·001
** Females n(%)**	281 (4·31)	129 (1·18)	<0·001
**Anti-HBc**			
** Number tested**	11,966	11,345	
** Proportion positive n(%)**	4583 (38·3)	1327 (11·7)	<0·001
** Males n(%)**	2541 (43·6)	759 (14·5)	<0·001
** Females n(%)**	1961 (33·0)	561 (9·2)	<0·001
**Anti-HBs**			
** Number tested**	10,037	10,403	
** Proportion positive n(%)**	6092 (60·7)	5763 (55·4)	<0·001
** Males n(%)**	3172 (62·1)	2474 (52·1)	<0·001
** Females n(%)**	2916 (59·2)	3279 (58·2)	0·280

^1.^ Any test.

* Sex is missing for two HBsAg positive individuals, one Indigenous and one non-Indigenous.

Odds ratios for HBsAg positivity were 4.08 (95% CI 3.54–4.71 *P*<0.0001) for Indigenous Australians versus non-Indigenous Australians, 1.53 (95% CI 1.42–1.66 *P*<0.001) for male versus female sex and 1.93 (95% CI 1.78–2.10 *P*<0.001) for living remotely versus in an urban centre.

### Birth cohort analysis

**Fig 2:**
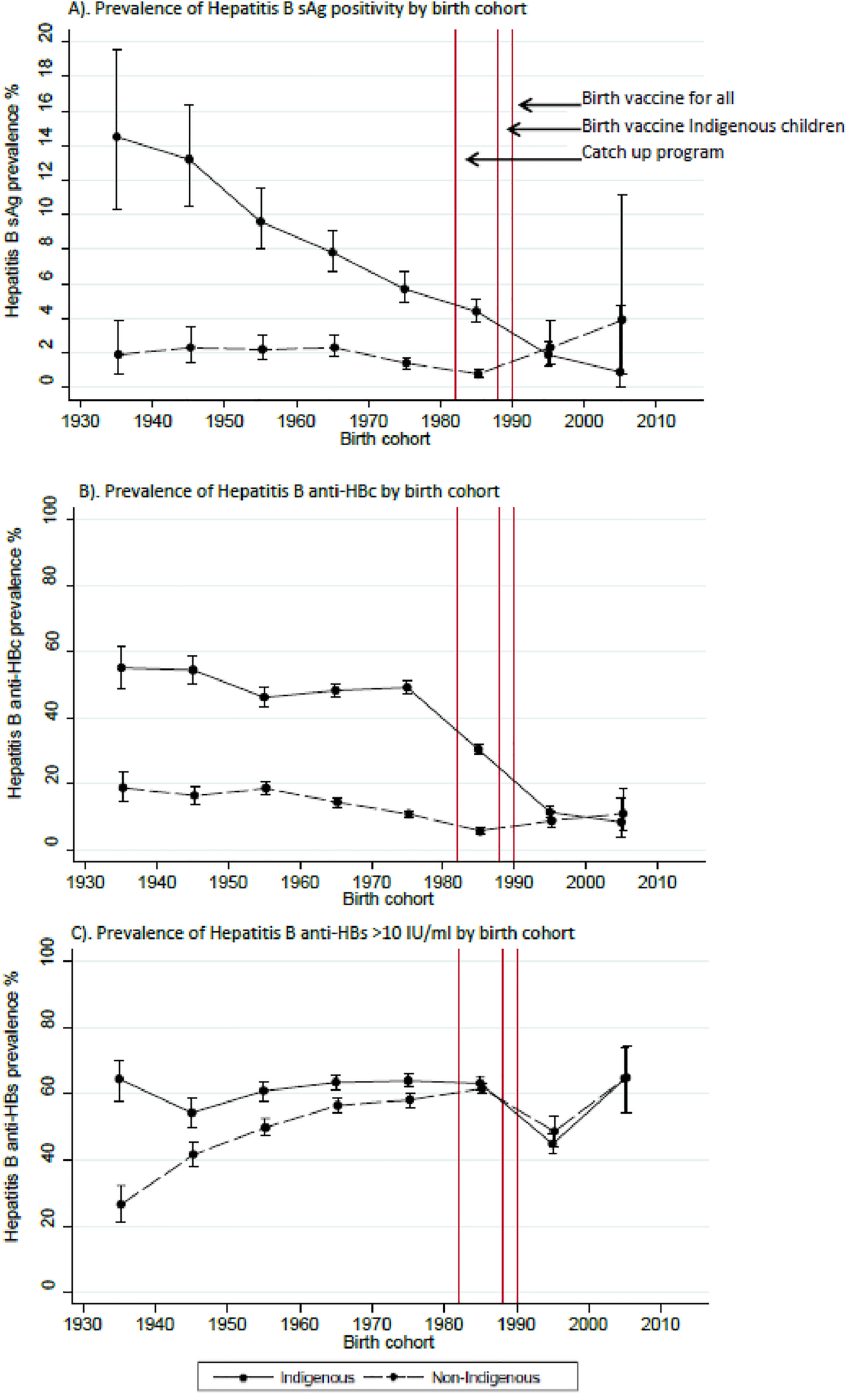
Graphs showing the prevalence (with 95% CI) of HBsAg (A), anti-HBc (B) and anti-HBs (C) by birth cohort for Indigenous and non-Indigenous people in the Northern Territory.

For Indigenous people, HBsAg positivity was much higher in early cohorts but decreased steadily with later birth periods to be similar to that of non-Indigenous people in the most recent cohorts (Fig 2A). For non-Indigenous people HBsAg positivity was around two percent for most birth cohorts, but increased after 1990 (Fig 2A).

Anti-HBc positivity was substantially higher for Indigenous than non-Indigenous people until the birth cohorts of individuals born after 1990, in which there was no difference (Fig 2B).

Anti-HBs positivity was also higher in Indigenous than non-Indigenous cohorts born between 1930 and 1979 (Fig 2C).

The trends of change in HBsAg, anti-HBc and anti-HBc was similar for males and females in the Indigenous and non-Indigenous populations (S2 Appendix).

**Fig 3:**
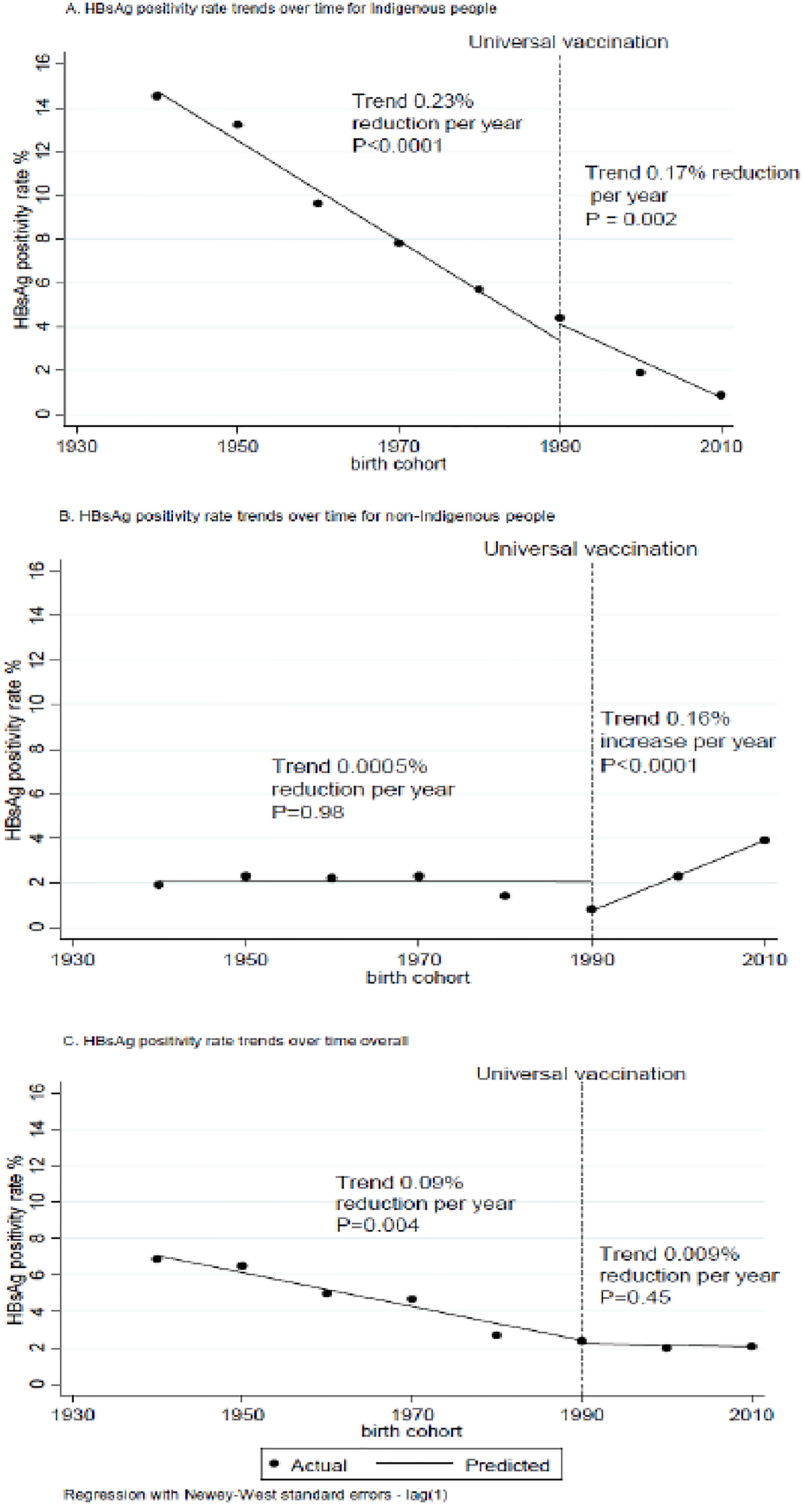
Interrupted time series analysis looking at HBsAg prevalence trends for (A) Indigenous, (B) non-Indigenous and (C) total population pre and post 1990 by birth cohort.

Interrupted time series analysis examining HBsAg positivity by birth cohort found that, for Indigenous people, the rate of decrease in positivity rates was not greater after the introduction of the universal vaccine than before (Fig 3A). In the non-Indigenous group there was an increase in HBsAg positivity rates post 1990 (Fig 3B).

Only 9.7% of those tested for any HBV marker between 2007 and 2011 were born in the universal vaccine era (post 1990). Of those born post 1990, HBsAg positivity was 1·89% (1·39–2·51) overall. Although it appears that in the post 1990 data there is a continued decrease in HBsAg positivity for Indigenous people and an increase in HBsAg positivity for non-Indigenous people (Fig 3A and 3B) the confidence intervals, particularly for the non-Indigenous group, are wide and overlapping.

## Discussion

This cross-sectional analysis of a large NT-wide HBV serology dataset documents a high contemporary prevalence of HBsAg positivity of 3·4% among people referred for HBV testing in the NT of Australia. HBsAg positivity was significantly higher in Indigenous Australians (6·08%) compared to their non-Indigenous counterparts (1·56%) and also in men (4·99%) compared to women (2·35%). These overall figures are higher than other recent Australian estimates of chronic HBV infection prevalence. The most recent Australian sero-survey documents an Australia-wide chronic HBV infection prevalence of 0·8% [17] and the National Australian Hepatitis B Mapping Project an Australia-wide chronic HBV infection prevalence of 1·02% and an NT prevalence of 1·68% [9]. A recent meta-analysis reported a chronic HBV infection prevalence in Indigenous Australians of 3·96% in those studies published since 2000 [1]. However, the majority of the data included in this paper came from antenatal testing, so it may not be representative of the overall Indigenous population.

It is encouraging that we document a significant reduction in HBsAg positivity over time in Indigenous individuals tested for HBV from 8·03% in the pre-vaccine era to 1·77% in the post 1990 period. However, it is important to note that the prevalence of HBsAg was falling prior to the introduction of universal vaccination with the rate of decline not significantly different in the post-intervention period. A data linkage study examining HBV seroprevalence in birthing mothers in the NT before and after the introduction of universal vaccination, reported HBV prevalence in Indigenous women falling from 3·5% in the pre-vaccine era to 0·8% in those born after 1989 [10]. Equivalent figures from our data are approximately double these estimates; this is attributable to our finding that Indigenous men have an OR of chronic HBV infection of 1·53 compared to Indigenous women. This disparity in HBsAg positivity between men and women has also been documented in other HBV-endemic regions of the world [18,19] and cautions against using antenatal data as a tool for assessing changes in chronic HBV infection prevalence over time. The Liu study [10] also suggests that chronic HBV infection prevalence was falling, albeit at a slower rate (reduction per year 0·08%) prior to universal vaccination.

There are several potential explanations for the decline in HBsAg positivity prior to the introduction of universal HBV vaccination. In the NT Indigenous population the exclusive HBV genotype identified to date is HBV/C4 [11]. Genotype C HBV-infected individuals undergo HBeAg sero-conversion at least a decade later than other genotypes [20]. Women of childbearing age with genotype C HBV typically remain HBeAg positive and have very high viral loads at the time they give birth, hence facilitating vertical transmission [21]. It is possible that changes in birthing practices over time, moving from community to hospital births (from the mid-1970s), may have contributed to this decline [22]. Cultural practices in general and specifically ‘men’s business’ (e.g. traditional circumcision, scarification, and bloodletting) have changed over time to potentially include less blood exposures [23,24]. The introduction of single use needles, syringes, and medication vials within public healthcare systems in the mid 1980’s and 1990’s respectively may have also contributed.

In non-Indigenous individuals HBsAg positivity actually increased post-1990. It is likely that this increase represents individuals who were not born in the NT and migrated from high prevalence countries, particularly China and South East Asia. The lack of country of birth data for the non-Indigenous people limits the ability to explore this group, however published evidence would suggest that at least half those with chronic HBV infection in the NT were born overseas [9]. Australian Bureau of Statistics 2011 census data show the absolute number of people in the NT from the Philippines and Cambodia doubled between 1996 and 2011, while those born in China, and India tripled [25]. We know from international data that migrants born overseas and now living in high-income countries, retain a chronic HBV infection prevalence that mirrors that of their birth country and that half are non-immune to HBV and would benefit from vaccination [26]. Obviously Australia’s universal childhood vaccination program will have no impact in this group and alternative screening and vaccination strategies need to be considered.

We report the first detailed data for the NT and for Indigenous Australians regarding anti-HBs and anti-HBc positivity, documenting high levels of anti-HBc positivity in Indigenous Australians: an overall prevalence of 38%, and > 50% in those born prior to 1950. These levels of anti-HBc positivity are similar to those reported from China prior to the introduction of their HBV vaccination program [27]. We also report a steady decrease in anti-HBc positivity over time in both Indigenous and non-Indigenous Australians, which predates the introduction of universal vaccination. However, anti-HBc positivity, representing natural infection, is still over 10% in the post-1990 birth cohort. We have previously described the molecular virology of HBV/C4 in detail [11], showing that it is a recombinant virus with a different serotype to the vaccine strain (ayw3 as opposed to adw2); this is a potential virological explanation for suboptimal vaccine efficacy.

Twenty year follow up data from The Gambia (also a region with a serotype mismatch) reports HBsAg positivity, in their cohort who were fully vaccinated at birth, as 0·8% and anti-HBc positivity as 27·4% [28]. This is similar to our data suggesting good protection from the vaccine program against chronic infection but only partial protection against having ever had natural infection. In contrast, data from Alaska, showed that in a vaccinated cohort followed up 22 years after initial vaccination zero individuals were HBsAg positive and only 1% of the cohort were anti-HBc positive [29].

The key limitation of this study is the use of testing data as opposed to a prospective serosurvey design. This methodology was guided by the wishes of NT Indigenous people to make the best use of existing data prior to asking for more blood samples, which as well as being culturally appropriate, had the additional benefit of being a low-cost approach. Although our denominator population was those referred for HBV serology, we believe it is representative of the NT population in general for the Indigenous population, where testing is largely driven by screening rather than clinical indication. In addition to antenatal screening, all Indigenous residents of the NT aged 15–55 years are offered an annual adult health check including HBV serology. Coverage of the Indigenous specific adult health check in the NT for 2011 was 20% [30] of the target population and we have serology results for > 20% of the NT Indigenous population in this age group. During the study time period there was minimal uptake of HBV antiviral therapy and HIV/HBV co-infection is rare [31]. Therefore at a population level there is unlikely to be an impact due to antiviral therapies on the results.”

Our data show the proportion of women tested in the childbearing years is greater than men, but that this difference is greater for non-Indigenous people. This suggests less true screening in the non-Indigenous group outside of antenatal testing and a greater role for clinically indicated testing. This may have led to an over-estimation of HBsAg positivity in non-Indigenous NT residents, particularly males. Our dataset lacks numbers in the very old and very young age groups, particularly for those born since the introduction of universal vaccination in 1990. It is likely that the potential referral bias will be most evident in these younger groups where there are small numbers. We do therefore caution against over-interpretation of the most recent data due to the relatively small numbers of tests carried out overall in the most recent birth cohorts (1990 onwards) and attendant wide confidence intervals around point estimates, particularly in the non-Indigenous group.

Furthermore, we may have over-estimated chronic HBV infection prevalence overall as we have assumed all HBsAg positive individuals have chronic HBV infection when some will represent acute HBV. We feel that this is a reasonable assumption based on our sensitivity analysis and the low rates of acute HBV in the NT (27 acute HBV notifications between 2007–2011[13]), equating to an absolute change of 0·17% on the overall HBsAg positivity rate of 3·4%.

In conclusion we report HBsAg prevalence in Indigenous Australians in the NT is falling but remains greater than 6%. Our data support the assertion that the HBV vaccination program is associated with a decreasing burden of chronic HBV infection; however they also suggest that the vaccine is only one factor responsible for this reduction in the NT. Our data identify an urgent need for systematic studies examining markers of HBV prevalence in people born after 1990 to enable a comprehensive analysis of the effectiveness of universal vaccination as an intervention. Our findings have implications not only for the planning and delivery of a sustainable public health response to chronic HBV infection in the NT, but for Indigenous people more widely. In addition, they have implications for other populations where a mismatch exists between currently used hepatitis B vaccines and dominant circulating HBV serotypes. The described low cost culturally acceptable model of providing contemporary surveillance data could be adapted to other Indigenous populations.

## Supporting information

S1 Appendix Sensitivity analysis used to determine the dataset for analysis. (DOCX)

S2 Appendix Graphs showing the prevalence of HBsAg (A), anti-HBc (B) and anti-HBs (C) by birth cohort for Indigenous and non-Indigenous people in the Northern Territory by gender. (TIFF)
