# Supplementary material for: Establishing contemporary trends in hepatitis B sero-epidemiology in an Indigenous population
Source: PLoS One. 2017 Sep 8;12(9):e0184082. doi: 10.1371/journal.pone.0184082 (PMC5590876; doi:10.1371/journal.pone.0184082)

**Appendix 1**

|  | | Overall | Indigenous | Non-­‐Indigenous |
| --- | --- | --- | --- | --- |
| **Median age in years at sample date (IQR)** | Whole group | 31.8 (24.6-­‐41.9) | 30.1 (21.5-­‐41.6) | 32.5 (26.1-­‐42.2) |
|  | 2007-­‐2011 | 32.4 (24.5-­‐43.7) | 30.8 (21.5-­‐43.3) | 33.2 (26.3-­‐44.0) |
|  | 2007-­‐2011 CHB only | 32.4 (24.5-­‐43.6) | 30.6 (21.5-­‐43.0) | 33.2 (26.3-­‐44.0) |
| **Sex Female**  **% (95% CI)** | Whole group | 58.3 (58.0-­‐58.7) | 55.4 (54.9-­‐56.0) | 59.9 (59.5-­‐60.3) |
|  | 2007-­‐2011 | 57.8 (57.3-­‐58.3) | 53.7 (52.8-­‐54.5) | 60.5 (59.9-­‐61.2) |
|  | 2007-­‐2011 CHB only | 58.0 (57.5-­‐58.5) | 54.0 (53.1-­‐54.8) | 60.6 (59.9-­‐61.3) |
| **HBsAg positive**  **% (95% CI)** | Whole group | 3.38 (3.25-­‐3.52) | 6.47 (6.17-­‐6.78) | 1.58 (1.46-­‐1.70) |
|  | 2007-­‐2011 | 3.4 (3.19-­‐3.61) | 6.08 (5.65-­‐6.53) | 1.56 (1.38-­‐1.76) |
|  | 2007-­‐2011 CHB only | 2.22 (2.06-­‐2.40) | 4.43 (4.25-­‐5.05) | 0.72 (0.60-­‐0.86) |
| **Anti-­‐HBs >10IU/ml**  **%(95%CI)** | Whole group | 61.4 (60.1-­‐61.8) | 62.5 (61.9-­‐63.2) | 60.3 (59.7-­‐61.0) |
|  | 2007-­‐2011 | 58.0 (57.3-­‐58.7) | 60.7 (59.7-­‐61.6) | 55.4 (54.4-­‐56.3) |
|  | 2007-­‐2011 CHB only | 58.5 (57.8-­‐59.1) | 61.4 (60.4-­‐62.3) | 55.8 (54.8-­‐56.7) |
| **Anti-­‐HBc positive**  **% (95% CI)** | Whole group | 27.0 (26.6-­‐27.3) | 42.5 (41.9-­‐43.2) | 12.7 (12.3-­‐13.1) |
|  | 2007-­‐2011 | 25.2 (24.7-­‐25.8) | 38.3 (37.4-­‐39.1) | 11.7 (11.1-­‐12.3) |
|  | 2007-­‐2011 CHB only | 24.2 (23.6-­‐24.7) | 37.3 (36.4-­‐38.2) | 10.6 (10.1-­‐11.2) |

**Table A1.1** Summary of median age, sex distribution, HBsAg, anti-­‐HBs and anti-­‐HBc data from each of the three analyses conducted as part of the sensitivity analysis.

Whole group = 88,175 individuals, 35% (30,464) Indigenous, 65% (57,711) non-­‐Indigenous

2007-­‐2011 = 35,633 individuals, 39% (14,025) Indigenous, 61% (21,608) non-­‐Indigenous

2007-­‐2011 CHB only = 35,287 individuals, 39% (13,823) Indigenous, 61% (21,464) non-­‐Indigenous

# A

Darwin urban Katherine Alice Springs

Remote Central Australia

Darwin rural Gove

Remote Top End

Pie chart showing usual residence of HBsAg positive individuals

20.4%

22.7%

1.5%

2.9%

6.1%

28.9%

17.6%

**B**

Darwin urban Katherine Alice Springs

Remote Central Australia

Darwin rural Gove

Remote Top End

Pie chart showing usual residence of HBsAg positive individuals

for those tested between 2007 and 2011 inclusive

16.7%

17.4%

1.4%

4.4%

6.5%

32.6%

21.2%

**C**

s

Darwin urban Katherine Alice Springs

Remote Central Australia

Darwin rural Gove

Remote Top End

Pie chart showing usual residence of HBsAg positive individual

for those tested between 2007 and 2011 inclusive

16.4%

14.4%

1.3%

3.5%

6.6%

37.6%

20.3%

**Figure A1.1** Pie charts showing usual residence of HBsAg positive individuals: A=whole group, B=2007-­‐2011, C=2007-­‐2011 CHB only.

| **HBsAg positive**  **prevalence** | **Male %**  **(95%CI)** | **Female %**  **(95%CI)** | **Odds Ratio**  **(95% CI)** | **P value** |
| --- | --- | --- | --- | --- |
| **Whole group**  **n=88,175** | 5.01 (4.76-­‐5.27) | 2.32(2.18-­‐2.47) | 0.45 (0.41­‐0.48) | <0.0001 |
| **2007-­‐2011**  **N=35,633** | 4.99(4.59-­‐5.40) | 2.35(2.13-­‐2.59) | 0.46 (0.41-­‐0.52) | <0.0001 |
| **2007-­‐2011**  **CHB** | 3.22 (2.90-­‐3.56) | 1.58 (1.40-­‐1.78) | 0.48 (0.42-­‐0.56) | <0.0001 |

**Table A1.2** Summary of HBsAg prevalence by sex with odds ratios for each of the three groups included in the sensitivity analysis.

|  | **Indigenous** | |  | **Non-­‐Indigenous** | |  |
| --- | --- | --- | --- | --- | --- | --- |
|  | Pre 1990  % (95%CI) | Post 1990  % (95%CI) |  | Pre 1990  % (95%CI) | Post 1990  % (95%CI) |  |
| **HBsAg** | 6.89  (6.57-­‐7.21) | 1.81  (1.30-­‐2.47) | P<0.0001 | 1.55  (1.43-­‐1.67) | 2.73  (1.84-­‐3.90) | P=0.002 |
| **Anti-­‐HBs** | 63.6  (62.9-­‐64.3) | 49.9  (47.5-­‐52.4) | P<0.0001 | 60.6  (60.0-­‐61.3) | 52.4  (48.9-­‐55.8) | P<0.0001 |
| **Anti-­‐HBc** | 45.4  (44.7-­‐46.1) | 12.1  (10.8-­‐13.6) | P<0.0001 | 12.7  (12.3-­‐13.1) | 12.3  (10.2-­‐14.5) | P=0.671 |

**Table A1.3** Summary of results in the pre and post vaccine era for the whole group analysis

|  | **Indigenous** | | **P value** | **Non-­‐Indigenous** | | **P value** | **Overall** | | **P value** |
| --- | --- | --- | --- | --- | --- | --- | --- | --- | --- |
|  | Pre 1990  % (95%CI) | Post 1990  % (95%CI) |  | Pre 1990  % (95%CI) | Post 1990  % (95%CI) |  | Pre 1990  % (95%CI) | Post 1990  % (95%CI) |  |
| **HBsAg** | 6.73  (6.25-­‐7.23) | 1.81  (1.21-­‐2.61) | <0.0001 | 1.52  (1.34-­‐1.72) | 2.49  (1.45-­‐3.95) | =0.0460 | 3.51  (3.29-­‐3.74) | 2.02  (1.47-­‐2.69) | =0.0002 |
| **Anti-­‐**  **HBs** | 62.6  (61.6-­‐63.6) | 46.4  (43.6-­‐49.2) | <0.0001 | 55.6  (54.7-­‐56.6) | 51.2  (47.1-­‐55.4) | =0.0424 | 58.9  (58.2-­‐59.6) | 47.9  (45.6-­‐50.3) | <0.0001 |
| **Anti-­‐**  **HBc** | 42.5  (41.6-­‐43.5) | 11.3  (9.7-­‐12.8) | <0.0001 | 11.8  (11.2-­‐12.4) | 9.6  (7.3-­‐11.9) | =0.0969 | 26.8  (26.2-­‐27.4) | 10.8  (9.5-­‐12.1) | <0.0001 |

**Table A1.4** Summary of results in the pre and post vaccine era for 2007-­‐2011 group

|  | **Indigenous** | | **P value** | **Non-­‐Indigenous** | | **P value** | **Overall** | | **P value** |
| --- | --- | --- | --- | --- | --- | --- | --- | --- | --- |
|  | Pre 1990  % (95%CI) | Post 1990  % (95%CI) |  | Pre 1990  % (95%CI) | Post 1990  % (95%CI) |  | Pre 1990  % (95%CI) | Post 1990  % (95%CI) |  |
| **HBsAg** | 5.00  (4.58-­‐5.45) | 0.72  (0.34-­‐1.28) | <0.0001 | 0.74  (0.62-­‐0.89) | 0.30  (0.04-­‐1.08) | 0.1860 | 2.36  (2.18-­‐2.55) | 0.59  (0.32-­‐1.01) | <0.0001 |
| **Anti-­‐ HBs** | 63.4  (62.3-­‐64.4) | 46.8  (44.0-­‐49.7) | <0.0001 | 56.0  (55.0-­‐56.9) | 52.1  (47.8-­‐ 56.3) | 0.0760 | 59.5  (58.7-­‐60.2) | 48.5  (46.1-­‐50.9) | <0.0001 |
| **Anti-­‐**  **HBc** | 41.5  (44.7-­‐46.1) | 10.5  (10.8-­‐13.6) | <0.0001 | 10.8  (10.2-­‐11.4) | 7.6  (5.6-­‐10.0) | 0.0119 | 25.7  (25.1-­‐26.3) | 9.7  (8.5-­‐11.0) | <0.0001 |

**Table A1.5** Summary of results in the pre and post vaccine era for 2007-­‐2011 CHB only group

**Figure A1.2** Graphs showing HBsAg, anti-­‐HBs and anti-­‐HBc prevalence patterns by birth using the whole group dataset.


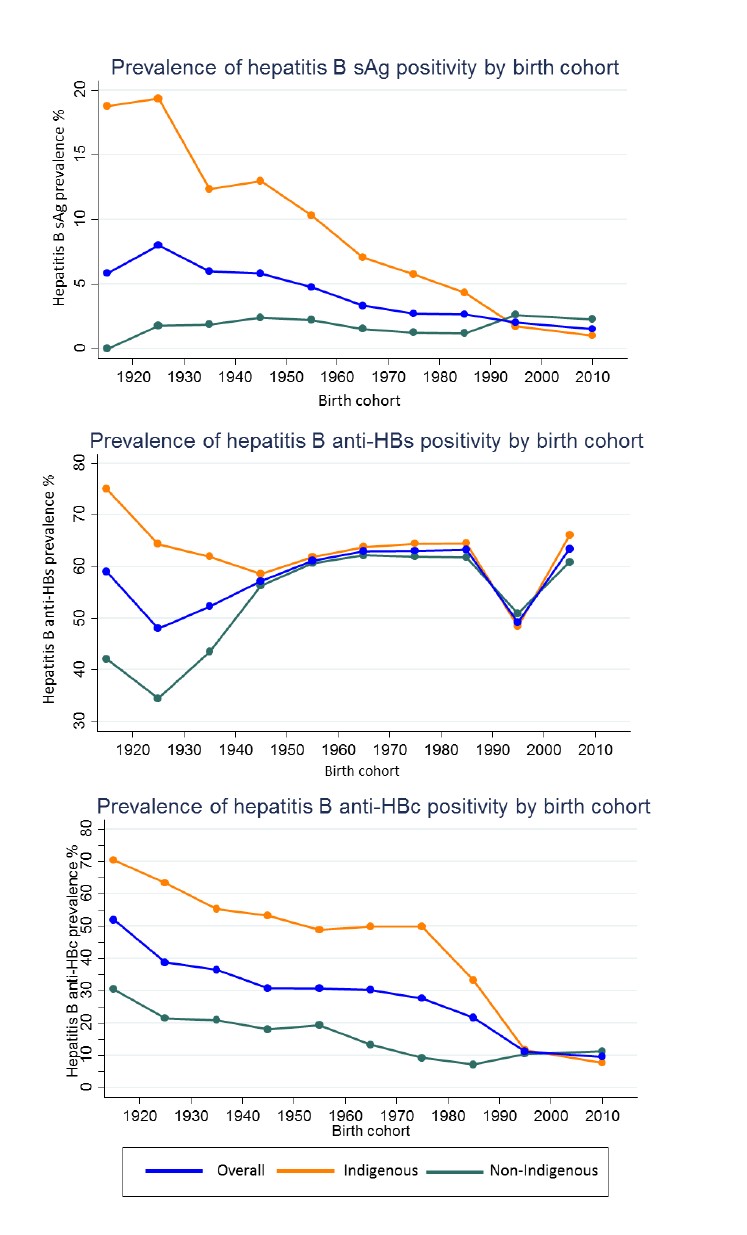


**Figure A1.3** Graphs showing HBsAg, anti-­‐HBs and anti-­‐HBc prevalence patterns by birth cohort using the 2007-­‐2011 CHB only dataset.


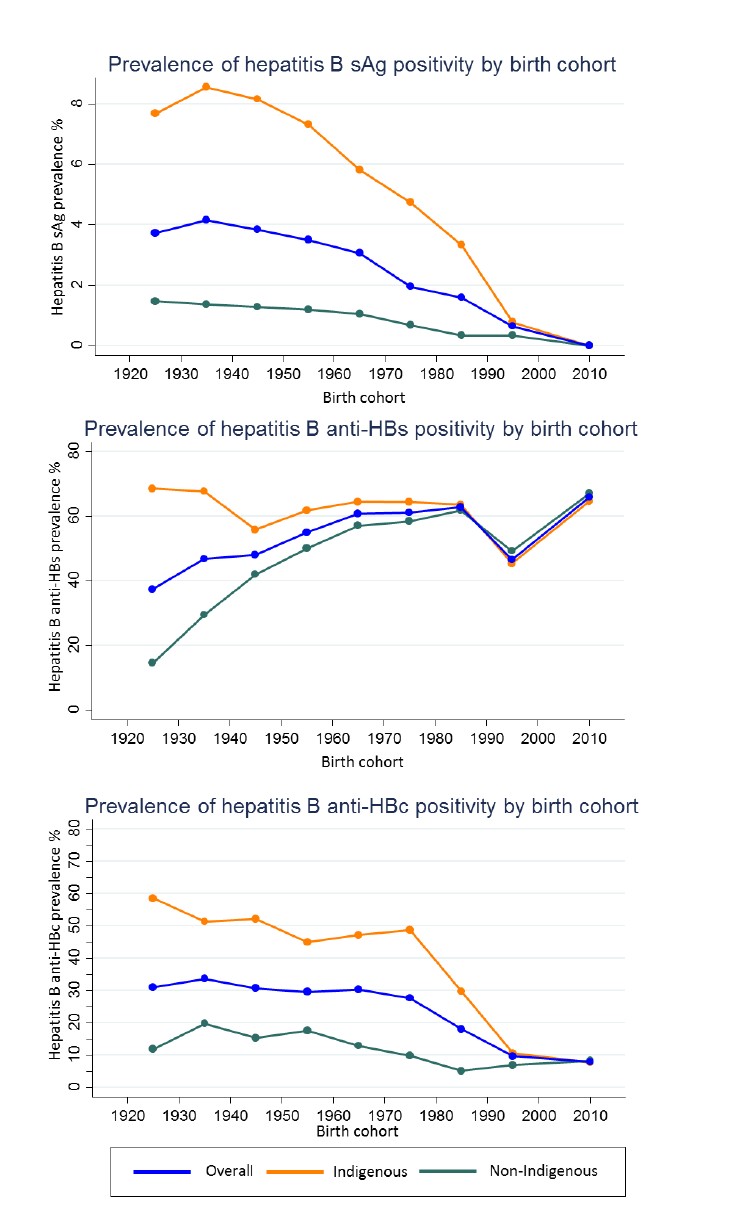


# Sensitivity analysis

**Figure A1.4** Graphs showing the birth cohort analysis for each of the three datasets used in the sensitivity analysis for HBsAg, anti-­‐HBs and anti-­‐HBc.


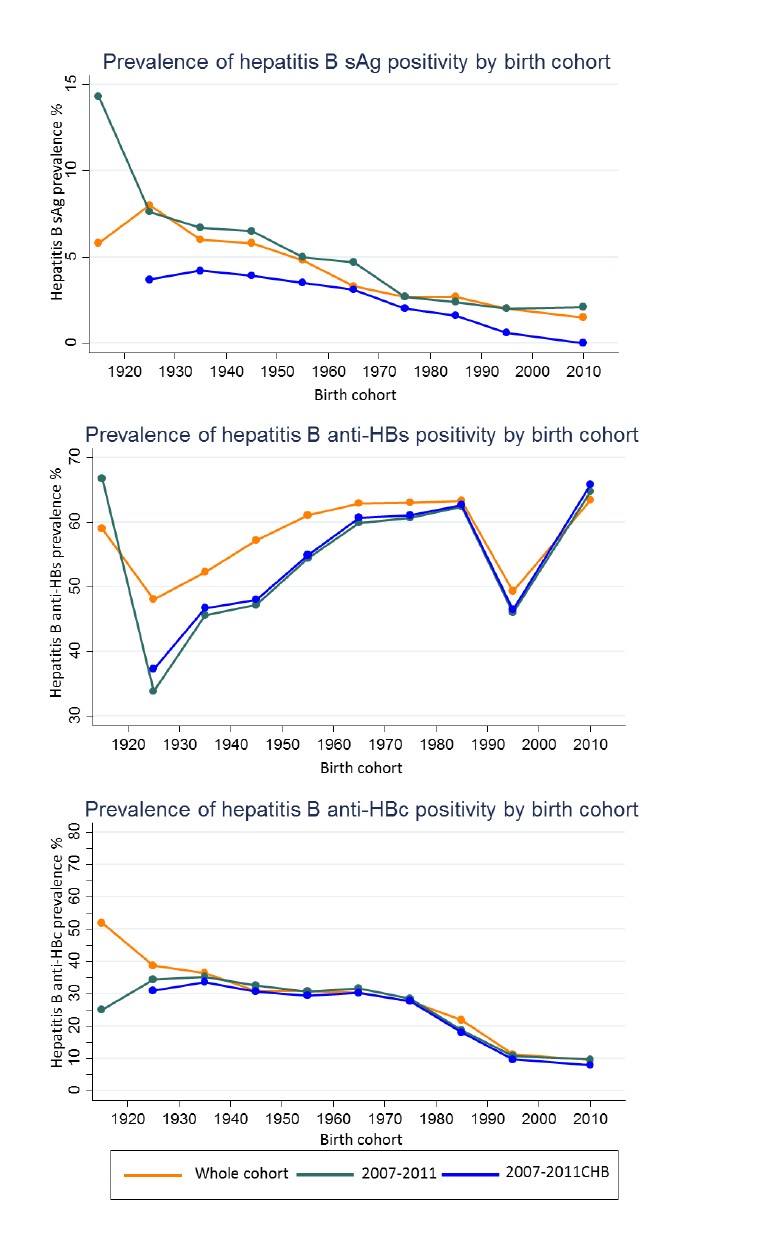

Supplement: S1 Appendix — (DOCX) [file pone.0184082.s001.docx]
