# Supplementary figures and images for: Establishing contemporary trends in hepatitis B sero-epidemiology in an Indigenous population
Source: PLoS One. 2017 Sep 8;12(9):e0184082. doi: 10.1371/journal.pone.0184082 (PMC5590876; doi:10.1371/journal.pone.0184082)

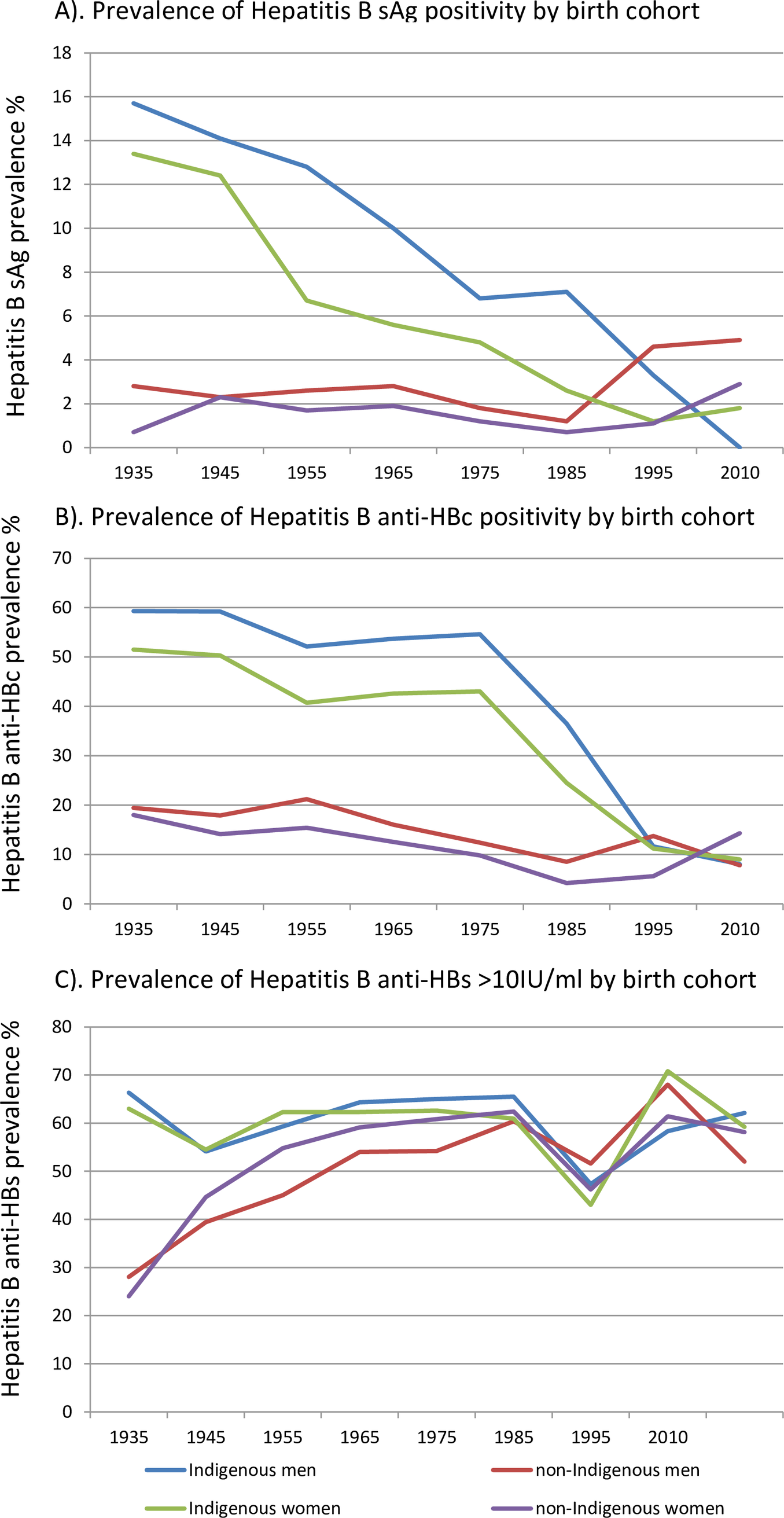

Supplement: S2 Appendix — (TIFF) [file pone.0184082.s002.tiff]
